# Gender bias in careseeking practices in 57 low– and middle–income countries

**DOI:** 10.7189/jogh.07.010418

**Published:** 2017-06

**Authors:** Janaína Calu Costa, Fernando C Wehrmeister, Aluísio JD Barros, Cesar G Victora

**Affiliations:** International Center for Equity in Health, Federal University of Pelotas, Pelotas, RS, Brazil

## Abstract

**Background:**

Preventive and curative medical interventions can reduce child mortality. It is important to assess whether there is gender bias in access to these interventions, which can lead to preferential treatment of children of a given sex.

**Methods:**

Data from Demographic and Health Surveys carried out in 57 low– and middle–income countries were used. The outcome variable was a composite careseeking indicator, which represents the proportion of children with common childhood symptoms or illnesses (diarrhea, fever, or suspected pneumonia) who were taken to an appropriate provider. Results were stratified by sex at the national level and within each wealth quintile. Ecological analyses were carried out to assess if sex ratios varied by world region, religion, national income and its distribution, and gender inequality indices. Linear multilevel regression models were used to estimate time trends in careseeking by sex between 1994 and 2014.

**Findings:**

Eight out of 57 countries showed significant differences in careseeking; in six countries, girls were less likely to receive care (Colombia, Egypt, India, Liberia, Senegal and Yemen). Seven countries had significant interactions between sex and wealth quintile, but the patterns varied from country to country. In the ecological analyses, lower careseeking for girls tended to be more common in countries with higher income concentration (*P* = 0.039) and higher Muslim population (*P* = 0.006). Coverage increased for both sexes; 0.95 percent points (pp) a year among girls (32.9% to 51.9%), and 0.91 pp (34.8% to 52.9%) among boys.

**Conclusion:**

The overall frequency of careseeking is similar for girls and boys, but not in all countries, where there is evidence of gender bias. A gender perspective should be an integral part of monitoring, accountability and programming. Countries where bias is present need renewed attention by national and international initiatives, in order to ensure that girls receive adequate care and protection.

Disaggregation of child health statistics by sex is important in order to identify gender bias in health intervention coverage, and in outcomes such as morbidity, mortality and nutritional status among children under the age of five years. Gender bias is a multidimensional social construct, in which different values are attributed to men and women in a given society, which can lead to preferential treatment of children of a given sex [1,2]; the use of this concept refers to a system of relations including sex, but goes beyond biological differences [3]. The study of gender bias in child health is affected by the greater biological vulnerability of boys compared to girls; in societies where there is no evidence of discrimination, boys show higher mortality rates than girls [4,5].

Two recent analyses assessed gender bias in the coverage of essential child health interventions in low– and middle–income countries (LMICs) [6,7]. Essential interventions may be classified as preventive (for example, measles vaccination, early initiation of breastfeeding, exclusive breastfeeding from 0–5 months, and use of insecticide treated bednets) or curative (use of antimalarials, careseeking for pneumonia, oral rehydration therapy, etc.) A UNICEF report showed no difference between girls and boys in terms of the seven interventions listed above [6]. There were also no differences in undernutrition (stunting, wasting or underweight). The numbers of countries included in these analyses ranged from 23 to 80 [6]. However, in spite of the lack of gender bias at national level, differences may exist at the subnational level, particularly among disadvantaged groups [8].

A recent systematic review investigated sex differences in hospitalizations for diarrhea, pneumonia and malaria in LMICs, and showed higher admission rates for boys, and higher case–fatality rates for girls [7]. However, hospital admissions are not a good indicator to study gender bias, because admission depends both on severity of the illness – which is likely to be greater for boys – and on careseeking by the caregivers [7]. Comparing careseeking rates among boys and girls for all cases of defined diseases or conditions is likely to be more useful in terms of detecting gender bias. In the same study, the authors analyzed data from 67 Demographic and Health Surveys (DHS) to investigate sex differences in careseeking by type of provider for diarrhea, fever, and pneumonia. Overall, more boys were taken to a health–care facility compared to girls [7].

Careseeking indicators are based on children who presented symptoms for each illness, usually in the two weeks before a survey. In these cases, the number of children is small, which leads to wide confidence intervals for these indicators, and may fail to detect differences between boys and girls as statistically significant due to low power [9].

We attempt to overcome this limitation by measuring sex differences using a composite careseeking indicator for three common childhood illnesses or symptoms. In addition, given the conflicting results of the two above–mentioned analyses, we expand our investigation to also assess whether these differences vary by wealth quintile, and whether sex differences in careseeking are associated with country characteristics such as income, religion and gender inequality indices. By doing so, we test the hypotheses that socioeconomic and related factors may modify the extent of gender bias in careseeking.

## METHODS

We analyzed data from nationally–representative Demographic and Health Surveys (DHS) conducted in low– and middle–income countries. We included all surveys with public–domain datasets available on the DHS website (http://dhsprogram.com/) as of May 2016, which had all the variables required for the analyses.

DHS asks mothers or caretakers of children under five years of age about diarrhea, fever, and symptoms of pneumonia (see Table S1 in **Online Supplementary Document(Online Supplementary Document)**). We used a composite careseeking indicator; the numerator was the number of children in a survey who were taken to an appropriate health care provider (defined by each country), during recent episodes of diarrhea, fever or suspected pneumonia, and the denominator was the number of children for which such an episode was reported during the two weeks preceding the interview. Pharmacies, shops and traditional practitioners were not considered appropriate providers.

The outcome variable was the proportion of children with symptoms who were taken to an appropriate provider. This was calculated separately for boys and girls in each survey, both at the national level and within each wealth quintile. Wealth indices were calculated for each survey through principal component analysis of household assets and building characteristics [10–12]. The first component resulting from the analysis was divided into quintiles, with Q1 representing the poorest, and Q5 the wealthiest, 20% of all families.

For the descriptive analyses, we selected the most recent survey from each country, from 2005 to 2014. Differences between the sexes in each country were assessed using chi–squared tests. Sex ratios were calculated for each survey by dividing careseeking proportions in girls and in boys, with values below 1.0 indicating gender bias against girls. The 95% confidence intervals for sex ratios were calculated using a jackknife approach based on repeated sub–sampling within the full survey sample. Interactions between wealth quintiles and sex of the child were assessed using Poisson regression with careseeking as the outcome.

Countries with more than one survey were included in the analyses of global time trends in careseeking between 1994 and 2014, using linear multilevel regression models with surveys as level one units and countries as level two units. We fitted separate trends for boys and for girls.

Ecological analyses were carried out with careseeking sex ratios as the outcome, based on the most recent survey for each country. The following explanatory variables were selected: region of the world according to UNICEF classification; religion (predominant and percentage in the population); country income groups; Gross Domestic Product *per capita* in USD; Gini coefficient of income inequality; and three indices related to gender equity (Gender Inequality Index, Gender Development Index, and Global Gender Gap Index) (see Table S2 in **Online Supplementary Document(Online Supplementary Document)** for full definitions and data sources) [13–20]. Associations between careseeking sex ratios and categorical explanatory variables were analyzed using analysis of variance (ANOVA), and those with continuous explanatory variables using Pearson´s correlation.

All analyses were carried out using Stata version 13.1 (StataCorp LP, College Station, Texas, USA), and considered the complex sampling structure of the surveys and the sampling weights.

## RESULTS

A total of 57 countries had DHS data sets since 2005 with the required variables. The median survey year was 2012. Sample sizes ranged from 1450 (Armenia) to 48 679 (India) children under five years (**Table 1**). The median sample size was 7526 children and the interquartile range was 5054 to 10 935.

**Table 1:** Characterization of 57 countries with available DHS surveys post–2005 according to region, income group, sample size and careseeking indicator

Country	Year	World Region (UNICEF)	Income group (World Bank)	Children under five years (n)	Children with diarrhea, fever or suspect pneumonia (n)			Careseeking sex ratio (CI 95%)		*P*–value
**Total**	**Boys**	**Girls**
Albania	2008	CEE & CIS	Upper middle	1586	267	145	122	0.9	(0.72–1.07)	0.292
Armenia	2010	CEE & CIS	Lower middle	1450	290	153	137	0.92	(0.66–1.19)	0.599
Azerbaijan	2006	CEE & CIS	Upper middle	2196	405	227	178	0.82	(0.57–1.06)	0.183
Bangladesh	2014	South Asia	Low	7567	3089	1614	1475	0.99	(0.84–1.15)	0.984
Benin	2011	West & Central Africa	Low	12 679	1857	954	903	0.96	(0.83–1.08)	0.521
Burkina Faso	2010	West & Central Africa	Low	13 716	4175	2143	2032	0.94	(0.88–1.00)	0.099
Burundi	2010	Eastern & Southern Africa	Low	7231	3713	1864	1849	0.96	(0.90–1.02)	0.302
Cambodia	2014	East Asia & Pacific	Low	6971	2248	1182	1066	1.09	(0.98–1.19)	0.076
Cameroon	2011	West & Central Africa	Lower middle	10 734	4443	2231	2212	0.93	(0.81–1.04)	0.263
Colombia*	2010	LAC	Upper middle	17 443	8669	4522	4147	0.93	(0.88–0.98)	0.020
Comoros	2012	Eastern & Southern Africa	Low	3022	951	489	462	0.95	(0.72–1.18)	0.683
Congo (Brazzaville)	2011	West & Central Africa	Lower middle	8857	3398	1733	1665	0.93	(0.82–1.04)	0.257
Congo D.R.	2013	West & Central Africa	Low	17 228	7292	3657	3635	1.01	(0.92–1.09)	0.781
Cote d’Ivoire	2011	West & Central Africa	Lower middle	7093	2453	1233	1220	1.03	(0.86–1.20)	0.687
Dominican Republic	2013	LAC	Upper middle	3606	1412	724	688	1.03	(0.91–1.15)	0.572
Egypt*	2014	Middle East & North Africa	Lower middle	15 466	5262	2867	2395	0.93	(0.89–0.97)	0.004
Ethiopia	2011	Eastern & Southern Africa	Low	10 808	3161	1621	1540	1.01	(0.85–1.17)	0.848
Gabon	2012	West & Central Africa	Upper middle	5747	2258	1135	1123	0.87	(0.72–1.02)	0.126
Gambia	2013	West & Central Africa	Low	7788	2127	1112	1015	0.96	(0.88–1.04)	0.370
Ghana	2014	West & Central Africa	Lower middle	5595	1396	767	629	1.03	(0.92–1.13)	0.513
Guinea	2012	West & Central Africa	Low	6424	2547	1311	1236	0.94	(0.83–1.05)	0.311
Guyana	2009	LAC	Lower middle	2105	600	315	285	1.06	(0.89–1.23)	0.427
Haiti*	2012	LAC	Low	6744	3650	1840	1810	1.11	(0.99–1.22)	0.044
Honduras	2011	LAC	Lower middle	10 592	4379	2335	2044	1.00	(0.93–1.06)	0.978
India*	2005	South Asia	Lower middle	48 679	11,336	6089	5247	0.93	(0.90–0.96)	0.000
Indonesia	2012	East Asia & Pacific	Lower middle	17 367	7029	3787	3242	0.96	(0.92–1.00)	0.068
Jordan	2012	Middle East & North Africa	Upper middle	10 128	3017	1595	1422	0.97	(0.88–1.07)	0.649
Kenya	2014	Eastern & Southern Africa	Low	20 093	7690	3922	3768	0.98	(0.94–1.03)	0.601
Kyrgyzstan	2012	CEE & CIS	Low	4247	392	200	192	0.88	(0.66–1.10)	0.320
Lesotho	2009	Eastern & Southern Africa	Lower middle	3606	1033	505	528	1.00	(0.89–1.12)	0.872
Liberia*	2013	West & Central Africa	Low	7058	3219	1659	1560	0.91	(0.83–0.98)	0.029
Madagascar	2008	Eastern & Southern Africa	Low	11 750	2029	1027	1002	0.92	(0.80–1.04)	0.244
Malawi	2010	Eastern & Southern Africa	Low	18 360	8227	4174	4053	0.99	(0.95–1.02)	0.634
Maldives	2009	South Asia	Upper middle	3761	1353	689	664	1.03	(0.96–1.09)	0.350
Mali	2012	West & Central Africa	Low	9582	1619	870	749	1.01	(0.83–1.19)	0.861
Moldova	2005	CEE & CIS	Lower middle	1533	368	172	196	0.96	(0.75–1.17)	0.723
Mozambique	2011	Eastern & Southern Africa	Low	10 291	2224	1131	1093	1.02	(0.93–1.10)	0.622
Namibia	2013	Eastern & Southern Africa	Upper middle	4818	1699	855	844	0.94	(0.86–1.03)	0.260
Nepal	2011	South Asia	Low	5054	1416	793	623	0.88	(0.75–1.01)	0.085
Niger	2012	West & Central Africa	Low	11 602	2852	1418	1434	0.93	(0.85–1.02)	0.165
Nigeria	2013	West & Central Africa	Lower middle	28 596	5787	2965	2822	1.00	(0.91–1.09)	0.897
Pakistan	2012	South Asia	Lower middle	10 935	5213	2750	2463	0.96	(0.93–1.00)	0.095
Peru	2012	LAC	Upper middle	9445	3134	1617	1517	1.02	(0.93–1.12)	0.543
Philippines	2013	East Asia & Pacific	Lower middle	7012	2413	1263	1150	1.01	(0.93–1.09)	0.707
Rwanda	2014	Eastern & Southern Africa	Low	7558	2190	1105	1085	0.99	(0.89–1.08)	0.903
Sao Tome and Principe	2008	West & Central Africa	Lower middle	1851	504	269	235	1.08	(0.90–1.26)	0.329
Senegal*	2014	West & Central Africa	Lower middle	6526	1633	865	768	0.76	(0.65–0.87)	0.000
Sierra Leone	2013	West & Central Africa	Low	10 618	3602	1797	1805	0.97	(0.91–1.02)	0.338
Swaziland	2006	Eastern & Southern Africa	Lower middle	2537	946	524	422	1.03	(0.90–1.16)	0.563
Tajikistan	2012	CEE & CIS	Low	4838	922	511	411	1.08	(0.93–1.22)	0.265
Tanzania	2010	Eastern & Southern Africa	Low	7526	2290	1163	1127	1.04	(0.96–1.12)	0.269
Timor–Leste	2009	East Asia & Pacific	Lower middle	9294	2661	1308	1353	0.98	(0.93–1.03)	0.473
Togo	2013	West & Central Africa	Low	6535	2262	1155	1107	1.07	(0.95–1.20)	0.221
Uganda*	2011	Eastern & Southern Africa	Low	7355	3946	2007	1939	1.04	(1.01–1.08)	0.008
Yemen*	2013	Middle East & North Africa	Lower middle	15 383	7345	3875	3470	0.85	(0.78–0.92)	0.000
Zambia	2013	Eastern & Southern Africa	Lower middle	12 714	4238	2139	2099	1.01	(0.95–1.06)	0.656
Zimbabwe	2010	Eastern & Southern Africa	Low	5203	1358	686	672	1.06	(0.91–1.21)	0.405

CI – confidence interval, CEE – Central and Eastern Europe, CIS – Commonwealth of Independent States, LAC – Latin America & the Caribbean

*Countries with significant sex differences in careseeking (*P* < 0.05).

**Figure 1:**
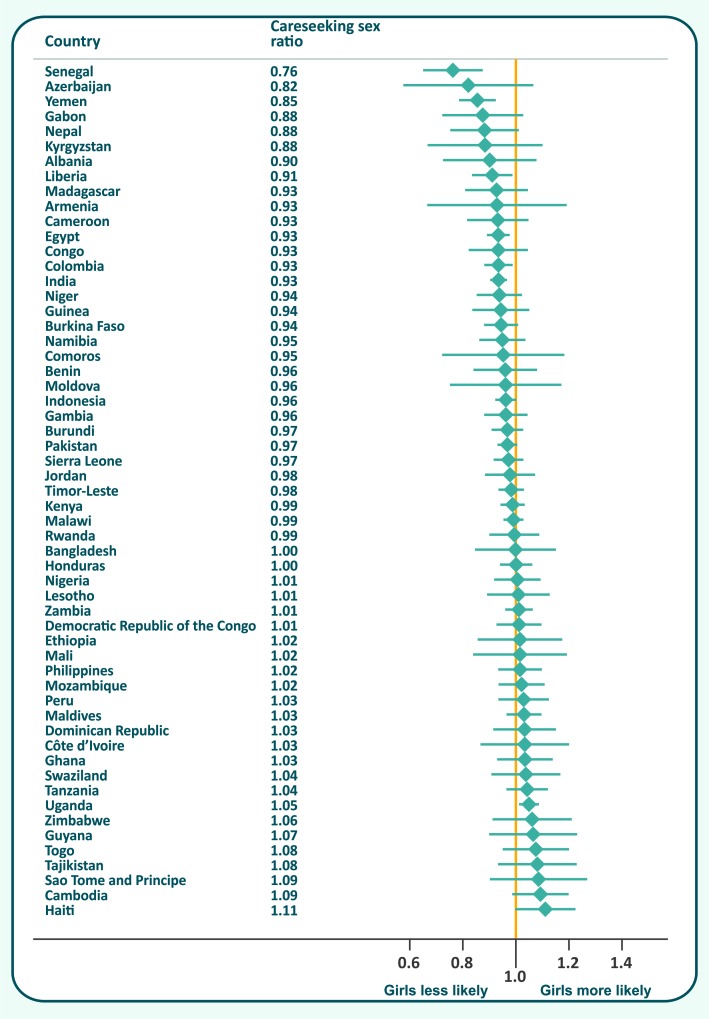
Careseeking sex ratios (95% confidence interval), by country.

Sex ratios for careseeking (girls/boys) ranged from 0.76 (0.68–0.85) in Senegal to 1.11 (0.99–1.24) in Haiti (**Figure 1**). The average value for all countries was 0.97 (0.96–1.00).

Eight countries showed statistical evidence of gender bias. In six of these (Senegal, Yemen, Liberia, Egypt, Colombia and India) girls were less likely to be taken to a provider, with sex ratios ranging from 0.76 to 0.94. In the other two countries, Haiti and Uganda (sex ratios of 1.11 and 1.05, respectively), girls were more likely to receive care. Further results at country level including 95% confidence intervals and p values are shown in **Table 1**.

**Figure 2:**
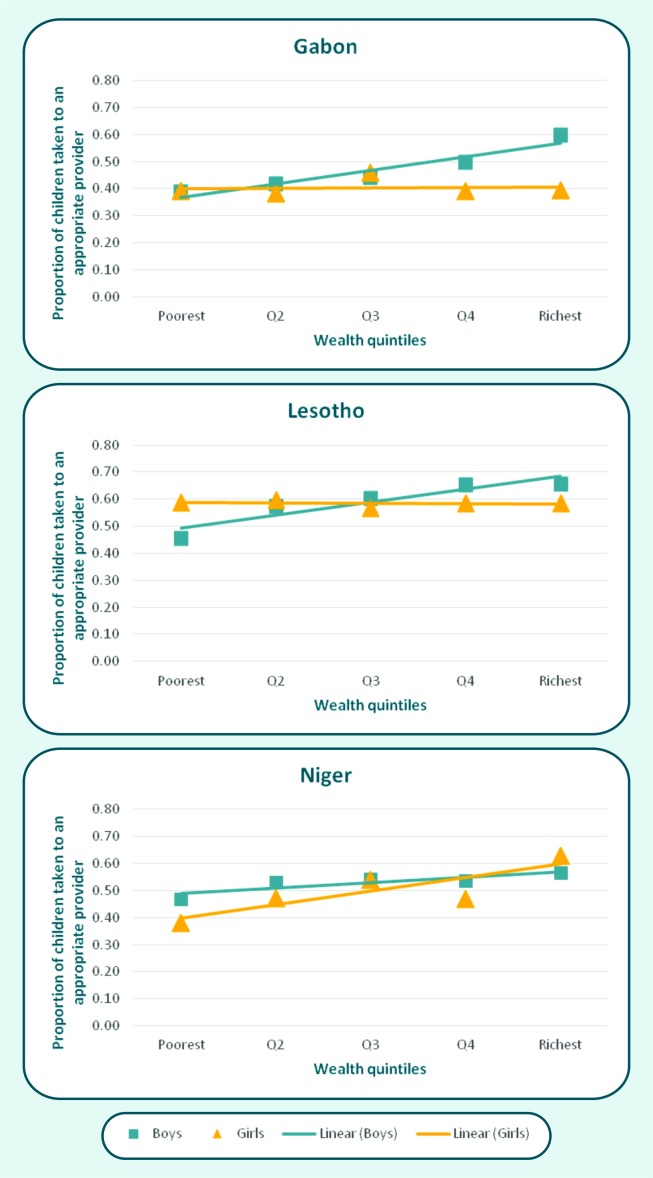
Careseeking for common childhood symptoms or illnesses by sex in countries with significant (*P* < 0.05) interactions between child sex and wealth quintile.

We also examined interactions between wealth and sex in careseeking coverage. Of the 57 countries, significant interactions (*P* < 0.05) were found in three. In Gabon and Lesotho, higher socioeconomic position was associated with greater careseeking for boys but not for girls; in Niger, the trend was in the opposite direction (**Figure 2**). Another four countries had interactions with p levels between 0.05 and 0.1: Burkina Faso, Congo Brazzaville, Dominican Republic and Senegal. Figure S1 in **Online Supplementary Document(Online Supplementary Document)** shows that interaction patterns were also inconsistent in these countries.

**Figure 3:**
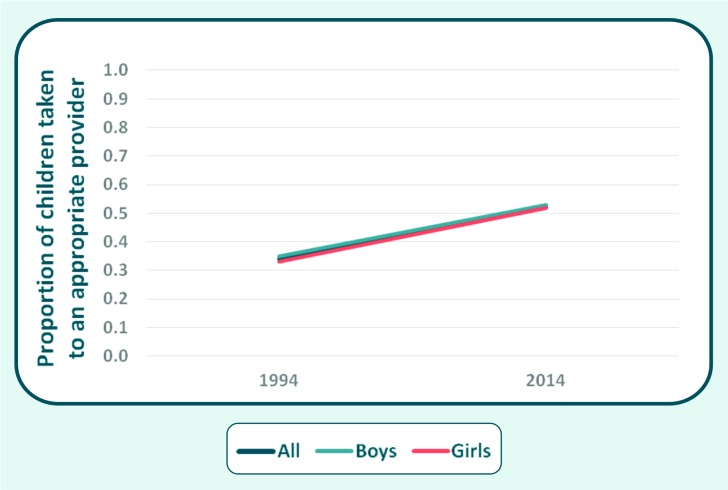
Gender differences in child health: evidences from Demographic and Health Surveys. Regression lines for changes in careseeking over time (1994–2014) by sex, for all countries combined.

Time trends analysis showed that global careseeking coverage increased by 0.93 percent point (pp) a year between 1994 and 2014 (from 33.9% to 52.4%) (**Figure 3**). Coverage increased for both sexes (*P* < 0.001): among girls the increase was 0.95 pp a year (32.9% to 51.9%), and among boys, 0.91 pp (34.8% to 52.9%).

Ecological analyses showed a lack of association between the careseeking sex ratio and most explanatory variables (**Table 2** and **Table 3**). There was no evidence of difference between the world regions. However, it should be noted that there are few surveys available for countries in South Asia and in Middle East & North Africa; most surveys are from countries in Eastern & Southern Africa, and in West & Central Africa.

**Table 2:** Ecological analyses of careseeking sex ratio and selected categorical exposure variables at national level

Variables	Categories	Number of countries	Mean	Standard deviation	*P*–value*
World region	CEE & CIS†	6	0.93	0.09	
	East Asia & Pacific	4	1.01	0.06	
	Eastern & Southern Africa	15	1.00	0.04	
	Latin America & Caribbean	6	1.03	0.06	0.055
	Middle East & North Africa	3	0.92	0.06	
	South Asia	5	0.96	0.06	
	West & Central Africa	18	0.97	0.08	
Country income group	Low	28	0.98	0.07	
	Lower–middle	20	0.98	0.06	0.398
	Upper–middle	9	0.95	0.08	
Gender Development Index groups‡	1. High equality	7	0.98	0.05	
	2. Medium to high equality	3	0.96	0.07	
	3. Medium equality	9	0.98	0.08	0.995
	4. Medium to low equality	8	0.98	0.05	
	5. Low equality	28	0.98	0.07	
Predominant religion	Christian	35	1.00	0.05	
	Muslim	19	0.94	0.07	0.011
	Other	3	0.97	0.11	

*P–value based on ANOVA.

†CEE & CIS: Central and Eastern Europe and the Commonwealth of Independent States.

‡Gender Development Index groups**:** Countries are divided into five groups by absolute deviation from gender parity in HDI values. Group 1: countries with high equality in HDI achievements between women and men (absolute deviation of less than 2.5%); Group 2: medium to high equality (absolute deviation of 2.5–5%); Group 3: medium equality (absolute deviation of 5–7.5%); Group 4: medium to low equality (absolute deviation of 7.5–10%); and Group 5: low equality (absolute deviation from gender parity of more than 10%) [20].

**Table 3:** Ecological analyses of careseeking sex ratio and selected continuous exposure variables at national level

Variables	Number of countries	Correlation (95% CI)	*P*–value*
GDP *per capita* (2012)	57	–0.257 (–0.485; 0.003)	0.053
GDP *per capita* – log (2012)	57	–0.190 (–0.429; 0.156)	0.157
Gini coefficient for income inequality	46	0.306 (0.018; 0.547)	0.039
Gender Inequality Index (2013)	50	0.074 (–0.208; 0.345)	0.607
Gender Development Index (2013)	50	0.090 (–0.193; 0.359)	0.531
Global Gender Gap Index (2014)	41	0.190 (–0.124; 0.470)	0.231
Muslim (% population)	57	–0.361 (–0.568; 0.111)	0.006
Christian (% population)	57	0.305 (0.049; 0.523)	0.021

CI – confidence interval

*P–value based on Pearson's correlation coefficient.

Regarding income levels, most of the countries surveyed are in the low– and lower–middle income groups, and no association was found between the level and careseeking sex ratios (**Table 2**).

There was a negative correlation, which was not statistically significant (*P* = 0.053) between continuous GDP *per capita* and the sex ratio, but not for log GDP per capita (*P* = 0.157).

None of the gender inequality indices were associated with the careseeking sex ratio (**Table 2** and **Table 3**). The Gender Development Index was tested both as a categorical variable, as recommended by its developers, and as a continuous index.

The religion variables were expressed both as categories of the predominant religion in each country (**Table 2**) and as the percent of Christians and Muslims in the population (**Table 3**). In both sets of ecological analyses, Christian religion was associated with improved care for girls, and Muslim religion with preferential careseeking for boys. These associations remained virtually unchanged after adjustment of the religion variables by GDP *per capita* (partial correlation coefficients of –0.351 for percent Muslim and –0.307 for percent Christian). Figure S2 in **Online Supplementary Document(Online Supplementary Document)** shows the careseeking sex ratios and 95% confidence intervals, for countries ranked according to the percentage of Muslim population.

We opted not to carry out extensive multivariable analyses because several explanatory variables are highly collinear (eg, GDI and income *per capita*, etc.) and because the gender indices also included socioeconomic variables in their construction.

## DISCUSSION

The analysis of the Demographic and Health Surveys, conducted in low– and middle–income countries, explored the magnitude of gender bias against girls, investigating whether families would be less likely to seek care from appropriate providers for girls with symptoms of fever, diarrhea or pneumonia, compared to boys. We expand upon the existing literature on this topic by calculating a new composite careseeking index encompassing three conditions – diarrhea, fever and suspected pneumonia – and therefore increasing the statistical power relative to earlier analyses in which each condition was treated separately.

We found evidence of gender bias in a limited number of countries. In contrast to the pervasive socioeconomic inequalities in careseeking and coverage, gender inequalities in careseeking are modest or even absent in most countries.

A systematic review explored studies on the recognition of signs and symptoms of, and/or careseeking for pneumonia, diarrhea or malaria in low– and middle–income countries. The authors identified seven publications that evaluated careseeking by sex; four which did not find significant differences between girls and boys, two reporting higher prevalence of careseeking for boys (in Burkina Faso and Indonesia), and one showing higher careseeking for girls, but only for malaria episodes [21]. The mixed results from this review are consistent with our analyses, which do not show a clear pattern of gender bias throughout the world.

At regional levels, we did not identify evidence of gender bias; however, in six countries careseeking was significantly higher for boys, and in two for girls. At the 5% *P* level, one would expect 1–2 significant pro–boy differences, and another 1–2 pro–girl differences, simply due to chance. We sought interactions between sex and wealth quintiles in careseeking for all 57 countries, but only detected significant interactions (with *P* < 0.10) in seven countries, which could have arisen by chance. In addition, interaction patterns were not consistent, sometimes with greater gender gaps in the wealthy, and for other countries with greater gaps among the poor.

The use of a composite careseeking indicator for three common conditions, using data from nationally representative surveys avoid small denominators – a frequent problem in analyses of careseeking – and thus increases statistical power [9]. Nevertheless, in our analyses sample size varied widely between surveys, and countries with the largest surveys such as India, results can be statistically significant even when absolute differences are small.

When comparing our results with the UNICEF analyses on careseeking for separate conditions, we found that three of the six countries we identified as presenting gender bias in the combined careseeking indicator had also been identified as such by UNICEF: Yemen (fever), Egypt (suspected pneumonia) and India (suspected pneumonia and diarrhea) [7]. It is important to highlight that the UNICEF report includes some unofficial health care providers that we did not include (such as shops and traditional practitioners), and that the year of the surveys may not be the same.

We used ecological analyses in an attempt to identify national characteristics associated with gender bias. Surprisingly, we did not detect correlations between careseeking sex ratios and gender inequality indices. A recent study reported a positive association between the Gender Inequality Index with under–five mortality rate for both sexes combined; this association remained after adjustment for GDP per capita, but separate associations with mortality rates for boys and girls were not investigated [22]. The authors speculate that if gender inequality is linked to maternal health, then mortality of boys and girls would be equally affected.

National levels of wealth were not associated with gender bias in careseeking, but bias was more likely in countries with unequal income distributions. We also found that religion was a cultural characteristic that explained part of the variability, with improved careseeking for boys in countries with a higher Muslim population. More research is needed to better understand the effects of religion and culture on careseeking, including whether the ecological association we report here is also found at individual level analyses within a given country, or whether it is due to an ecological fallacy.

Other limitations in the data should be recognized. Differences in careseeking could be due to increased severity of infectious diseases among boys [7], but our results showing similar careseeking rates in most countries suggest that this did not bias the results. Also, information on the incidence of illness and on careseeking patterns is based on maternal recall, which may or may not vary systematically according to child’s sex [21].

In addition, a composite index for careseeking does not reflect how different illnesses may be perceived along the spectrum of severity; more detailed analyses might consider only severe cases (such as bloody diarrhea, for example) but this would further reduce the denominator and analyses would only be possible for very large sample sizes.

Lastly, our analyses are limited to the most recent survey per country, so that results on time trends must be interpreted with caution as for some countries the most recent publicly available survey was carried out a decade or more ago, as is the case for India.

## CONCLUSIONS

Our results suggest that, with a few exceptions, the overall frequency of careseeking for common health conditions is similar for boys and girls in most, but not in all countries. Similar results are available for under–five mortality [4,7,23]. Countries where there is evidence of gender bias in careseeking need renewed attention of national and international initiatives, in order to ensure that girls receive adequate care and protection. In addition, more research is needed to understand the reasons behind the different treatment for girls and boys in these circumstances, including a mixture of qualitative and quantitative methods.
