# Supplementary material for: Gender bias in careseeking practices in 57 low– and middle–income countries
Source: J Glob Health. 2017 Jun 4;7(1):010418. doi: 10.7189/jogh.07.010418 (PMC5460396; doi:10.7189/jogh.07.010418)

Online Supplementary Document

Calu Costa et al. Gender bias in careseeking practices in 57 low– and middle–income countries

J Glob Health 2017;7:010418

Supplementary Table 1 - Careseeking questions in DHS questionnaires.

Indicator	Numerator	Questions	Denominator	Questions	Comments
**Careseeking for suspected pneumonia**	Sought treatment from a health facility or provider, excluding pharmacy, shop and traditional practitioner.	Did you seek advice or treatment for the illness from any source?	Alive children, 0-59 months, with suspected pneumonia in the past 2 weeks	Has (NAME) had an illness with a cough at any time in the last 2 weeks?	New definition of suspected pneumonia: short and rapid breaths AND problem in the chest.<br><br>Old definition: short and rapid breaths
		Where did you seek advice or treatment?		When (NAME) had an illness with a cough, did he/she breathe faster than usual with short, rapid breaths or have difficulty breathing?	
		Anywhere else?		Was the fast or difficult breathing due to a problem in the chest or to a blocked or runny nose?	
**Careseeking for diarrhea**	Sought treatment from a health facility or provider, excluding pharmacy, shop and traditional practitioner.	Did you seek advice or treatment for the diarrhea from any source?	Alive children, 0-59 months, with diarrhea in the past 2 weeks.	Has (NAME) had diarrhea in the last 2 weeks?	
		Where did you seek advice or treatment?			
**Careseeking for fever**	Sought treatment from a health facility or provider, excluding pharmacy, shop and traditional practitioner.	Did you seek advice or treatment for the illness from any source?	Alive children, 0-59 months, with fever in the past 2 weeks	Has (NAME) been ill with a fever at any time in the last 2 weeks?	
		Where did you seek advice or treatment? Anywhere else?			

Supplementary Table 2 – Ecological exposures definitions

Variable	Description	Source
World region	UNICEF classification: Central and Eastern Europe and the Commonwealth of Independent States (CEE & CIS); East Asia and the Pacific, Eastern and Southern Africa, Latin America and Caribbean, Middle East and North Africa, South Asia, West and Central Africa.	UNICEF
Predominant religion	The countries were classified based on the predominant religion in Christian, Muslim and Other and according to the percentage of each religion in the population.	*The World Factbook – Central Intelligence Agency* (CIA)
Income group	Income groups are defined based on Gross National Income (GNI) *per capita*, calculated using the World Bank Atlas method. Low-income economies: US$1,045 or less; middle-income economies are those with a GNI *per capita* of more than $1,045 but less than $12,736. Lower-middle-income and upper-middle-income economies are separated at a GNI *per capita* of $4,125.	World Bank
Gross Domestic Product *per capita*	GDP is gross domestic product converted to international dollars using purchasing power parity rates (PPP). An international dollar has the same purchasing power over GDP as the U.S. dollar has in the United States. GDP at purchaser's prices is the sum of gross value added by all resident producers in the economy plus any product taxes and minus any subsidies not included in the value of the products. It is calculated without making deductions for depreciation of fabricated assets or for depletion and degradation of natural resources. Data are in current international dollars based on the 2011 International Comparison Program (ICP) round.	World Bank
Gini coefficient of income inequality	Measure of the deviation of the distribution of income among individuals or households within a country from a perfectly equal distribution. A value of 0 represents absolute equality, a value of 100 absolute inequality.	World Bank
Gender Inequality Index (GII)	A composite measure reflecting inequality in achievements between women and men in three dimensions: reproductive health, empowerment and the labour market.	UNPD
*Gender Development Index (*GDI)	GDI measures gender gaps in human development achievements by accounting for disparities in three basic dimensions: health, knowledge and living standards using the same component indicators as in the Human Development Index (HDI). The GDI is the ratio of the HDIs calculated separately for females and males using the same methodology as in the HDI. It is a direct measure of gender gap showing the female HDI as a percentage of the male HDI.	UNPD

Global Gender Gap Index (GGGI)	The GGGI examines the gap between men and women in four fundamental categories: Economic Participation and Opportunity, Educational Attainment, Health and Survival and Political Empowerment. The final value ranges between 1 (equality) and 0 (inequality), thus allowing for comparisons relative to ideal standards of equality in addition to relative country rankings.	World Economic Forum

Supplementary Figure 1 – Careseeking for symptoms of common symptoms/illnesses in childhood in countries with significant (0.05<p<0.10) interactions between child sex and wealth quintile.

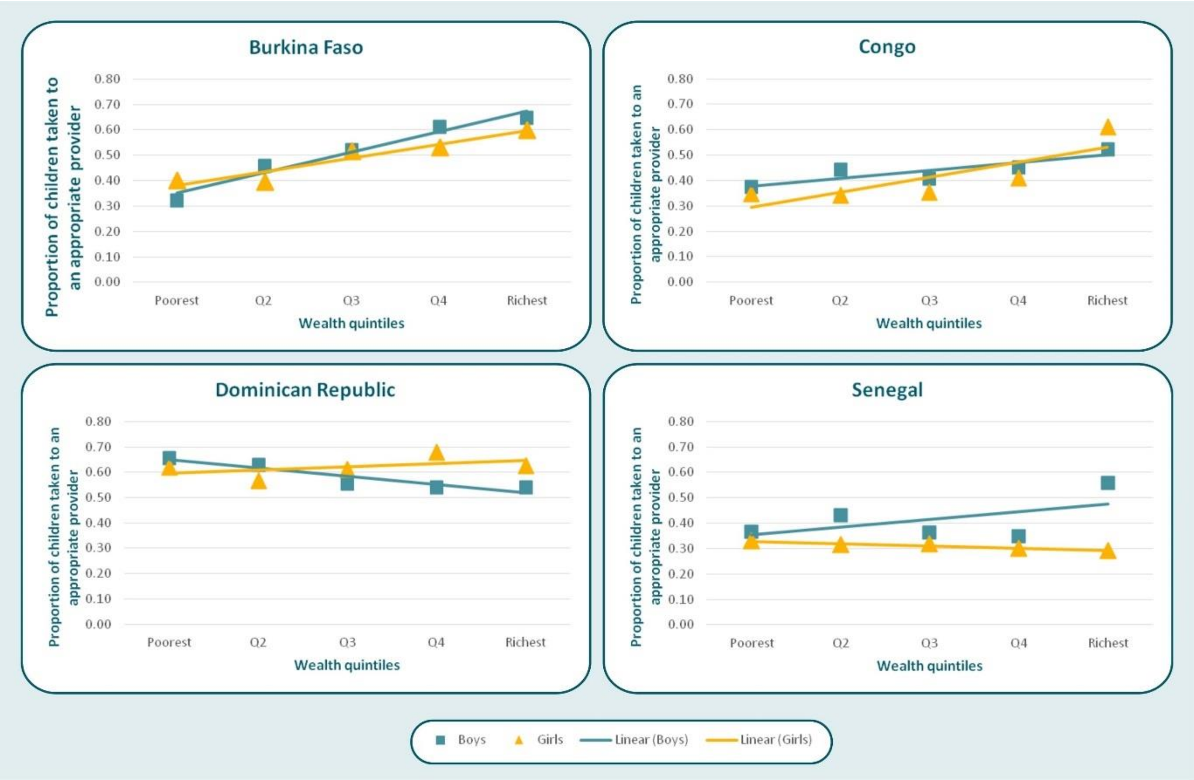

Supplementary Figure 2 – Percentage of Muslim population and careseeking sex ratios (95% CI), by country.

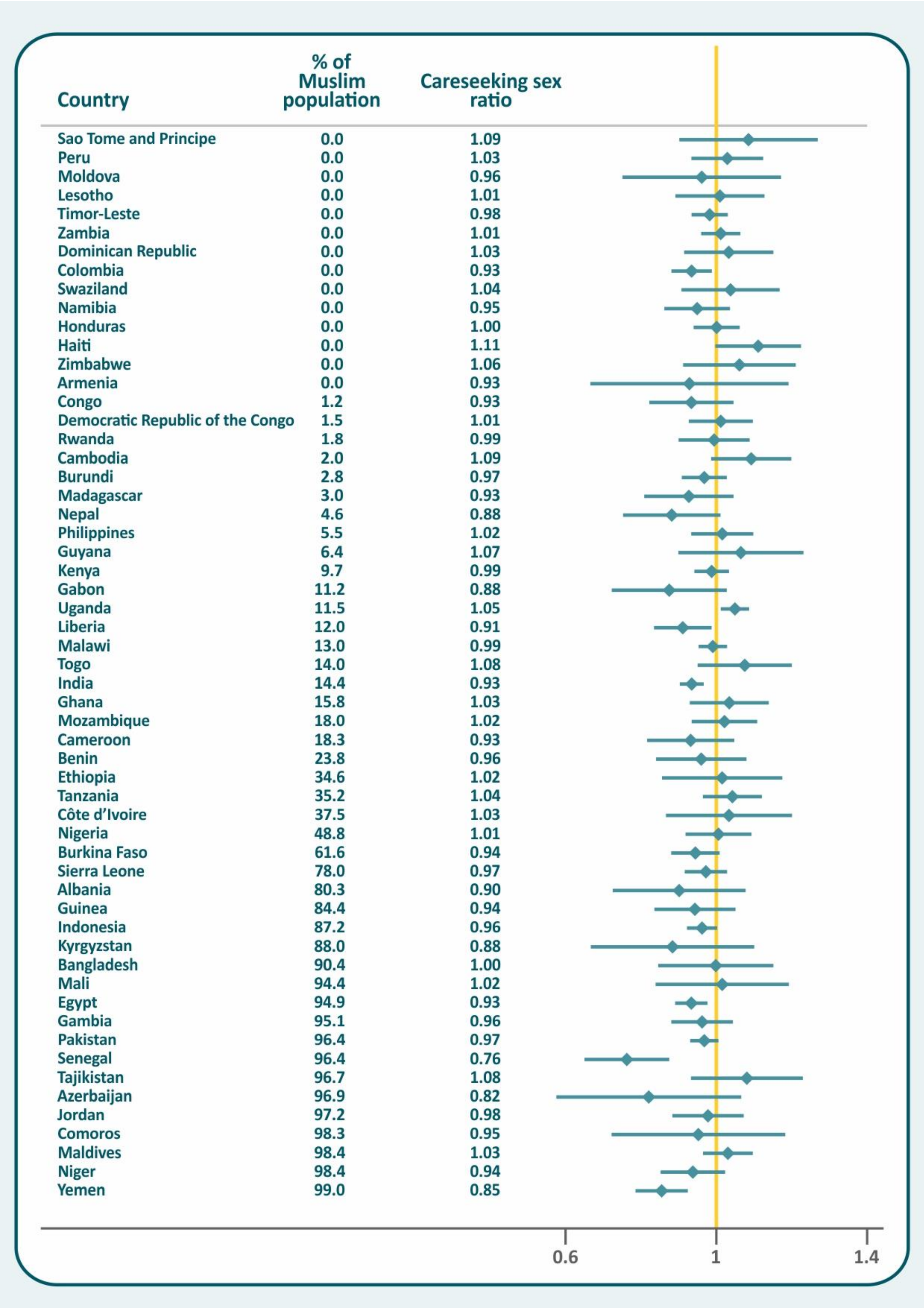

Supplement: Online Supplementary Document [file jogh-07-010418-s001.pdf]
